# Treatment of Pulmonary Phthisis

**Published:** 1893-07-08

**Authors:** 


### ROYAL HOSPITAL FOR DISEASES OF THE CHEST, CITY ROAD, E.C.*

### Treatment of Pulmonary Phthisis.

The first point in the general treatment is the hygiene of the ward, and without professing to have much that is new to offer, it may not be out of place to mention a few details on which stress is laid in this hospital.

*Ventilation* is always a difficult matter, and we are far from having solved the problems it offers. Still something has been done, and of this we will give a brief account. As the wards face the noisy City Road double windows are a necessity, and thus a ventilating shaft bringing up a current of fresh air can be made to open into the space between the windows without causing a direct draught on the beds near. When the new wing was built six or seven years ago an elaborate scheme was devised for ventilating the wards by means of currents of hot air in shafts in the thickness of the walls, having inlets near the ceilings, but, unfortunately, the scheme has not quite realised the hopes of its inventor, and it is now not in regular use. Ventilation is further helped by openings from the wards into the long and lofty corridors running the the whole length of the building, with windows at each end. But we are still looking for the perfect system. Of minor details, curtains to the beds, as being harbourers of dust, are forbidden, except for those which, being close to the door, are liable to occasional draughts. The spittoons all contain a solution of carbolic acid (1 in 40), and great care is taken in carrying out the order forbidding patients to expectorate into their handkerchiefs.

*Diet* must, of course, play a very important part in hygienic treatment, and in respect to this the patients are treated on a distinctly liberal scale—a very necessary condition in dealing with persons who have generally had a difficulty in getting enough of even the simplest food. Meals are taken four times a day, and it is the rarest possible occurrence to have any grumbling either over the quality or quantity of the food supplied.

In one of the earliest annual reports of this hospital —viz., that for 1826—the great advantage insisted on is that "the wards are kept day and night at a moderate summer temperature," and this condition still plays an important part in the general treatment of our patients. By a carefully-managed system of hot-water pipes all parts of the building are well warmed, and the comfort of this arrangement is much appreciated by all the patients.

One other point may here be alluded to, which, though apparently trivial, really is of great importance, —we refer to the provision of an airy recreation-room, furnished with games, books, writing materials, &c., and in which during the early part of the afternoon the women can sit and gossip or otherwise amuse themselves, while later, the men much appreciate the enjoyment of a quiet pipe.

In dealing with phthisical patients, one is bound to feel how large a part the above details play in improving the general health, and that after all treatment by drugs is only secondary in importance. But though secondary, certain drugs do assist in a very marked way, in building up an enfeebled constitution, or in alleviating various distressing symptoms.

Probably cod liver oil (in doses of ʒii. twice a day), and extract of malt (same dose as of oil), should take the first place among drugs directed to the general constitutional treatment, and were it not for the extraordinary fondness for taking medicines which most patients exhibit, there are many cases which really could be treated by these drugs alone, some linctus or lozenges being given occasionally for the cough. As it is, one has to prescribe tonics in one form or another—such as

* By the Resident Medical Officer.

nux vomica (♏ x.) with a mixture containing carbonate of ammonia (gr. iv.), and spirit of chloroform (♏ xx.); or strychnine (♏ iii. or iv.), with or without dilute phosphoric acid (♏ x. or xv.).

Some patients do well on arsenic, especially in an alkaline mixture (liq. arsenic, ♏ ij., pot. bicarb. gr. xv., aq., ad. ℥i). The vegetable bitters, too, are much used, —gentian in an alkaline mixture (pot. bicarb. gr x., tinct. zingib. ♏ i., infus. rhei. ʒi., infus. gent. co. ad. ℥i.), with which nux vomica is generally combined; and calumba, given also with pot. bicarb. and tinct. nux vom. For anæmic patients iron is frequently used, either combined with strychnine (ferr. et quin. citr. gr. v., liq. strychn. ♏ iij.) or the citrate is given alone, or liq. ferri perchloridi is given in doses of ♏ xx. with spirit of chloroform (♏ x.) and infusion of quassia (ad. ℥j.)

Coming now to the different symptoms of phthisis and their treatment, the most important and the most difficult to treat is cough, which will be discussed in a separate paper, together with expectoration. Other symptoms are (1) fever, (2) night sweating, (3) sickness and dyspepsia, (4) diarrhœa, (5) hæmoptysis, (6) pains in the chest, (7) sleeplessness.

1. *Fever* occurs both in the acute early stage of phthisis and in the later stages with cavitation, and its treatment must be varied in accordance with the conditions it accompanies. In the early stage rest in bed is the main point, with light diet and saline mixtures to keep the skin acting well. With an evening temperature ranging up to 103 deg. F. and above we begin with tepid sponging, which generally proves successful. In the event of failure with this simple means, either antipyrin or phenacetin is used, generally the latter, five or ten grains of which are given about five p.m., and the temperature taken at six and eight p.m. If the temperature is still up a second dose is given, and this rarely, if ever, fails to bring the temperature down satisfactorily. With young people phenacetin occasionally acts rather strongly (thus a temperature may come down from 104 deg. to 97 deg. in less than an hour with startling suddenness), and care must be taken especially in giving the first few doses.

For the high temperature of later stages phenacetin is the drug most in favour, and sponging has less value. Quinine in doses of three to five grains, given three times a day, is frequently used, and has given fair results, though not so good as those obtained by the use of phenacetin. Inhalations of steam from water at 140 deg. F., to which thirty minims of liquid carbolic acid have been added, are used when there is much cavitation, and act very well, presumably by checking the suppuration going on in the lung.

2. *Night Sweating.*—This is usually treated by the zinc and belladonna pill (zinc. ox. gr. iij., ext. belladonn. gr. $\frac{1}{4}$, ext. hyoscy. gr. i.), or a pill containing atropine gr. $\frac{1}{200}$. These are given about eight p.m., and usually check the sweating very rapidly. Sponging with water and vinegar is sometimes tried, but is of much less value than the above pills.

Hypodermic injections containing morphia gr. $\frac{1}{8}$, and atropine gr. $\frac{1}{200}$ are given to relieve sweating, and also to induce sleep at the same time.

3. *Sickness and Dyspepsia.*—The vegetable bitters (see above) answer very well in these cases, provided the cause of the want of appetite, &c., is not to be found in over-doses of morphia, taken before admission by patients, in the form of linctus, where the remedy is obvious. Tincture of calumba is especially valuable in checking the vomiting sometimes met with independently of coughing; it is given in doses of ♏ xx or ♏ xxx in a little water as soon as the feeling of nausea comes on. Mixtures containing the carbonates of bismuth and magnesia (gr. x. of each) with or without a few minims of dilute hydrocyanic acid are frequently given when there is evidence of gastric irritation and an effervescing mixture has been of great service against vomiting.

4. *Diarrhœa* is, of course, a very troublesome symptom, but will often yield to a Bismuth mixture, of which ℥ss. is given after each motion with seven to ten minims of tincture of opium, or a mixture is given containing liq. calc. ʒiii., tinct catechu. ♏ xxv., and decoct. hæmator. ad. ℥i., to which tinct. opii. is added. In other cases the hospital pil. cupr. sulph. c. opio (cupr. sulph. gr. ss. pulv. opii gr. $\frac{1}{4}$, ext. gentian. q.s.) proves more satisfactory given three times a day. Finally, starch and opium enemata are very useful, the more so as ulceration is mostly found in the colon.

5. *Hœmoptysis.*—The ordinary methods of perfect quiescence on a mattress without a pillow, ice to suck, no stimulant, no solid food, and very little liquid are often sufficient. To aid these, mist ergotæ co. is sometimes given (containing ext. ergot. liq. ♏ xxv., and tr. hamamel. ♏ xx) with only moderate results; and on the whole more success is obtained by giving as suggested in Dr. Andrew's Harveian oration of 1890, the tincture of aconite; usually tr. aconit. ♏ i, with tr. opii ♏ ii or iij, in a little water every hour up to five, six, or eight doses, then less often. Hypodermic injections of morphia have also proved very efficient. The mental condition in such cases is a matter of great importance, and care is always taken to endeavour to raise the patient's spirits and cheer him up, as usually he is dreadfully frightened, and the heart's action greatly quickened and disturbed.

6. *Pains in the Chest* are, as a rule, pleuritic, and hence are frequently at once relieved by strapping the affected side firmly by strips of plaster reaching from the sound side of the sternum round the affected side to the sound side of the spine. In certain cases, as for instance, when both sides are affected, or when breathing is very difficult, this method is inadmissible and other plans are tried, such as hot fomentations, painting with iodine (equal parts of liniment and tincture) or small blisters. One or other of these means rarely fails, strapping being on the whole that most commonly used.

7. *Sleeplessness* is, of course, often due to pain or to a tiresome cough, but in certain cases seems to be quite independent of these. For such patients hypodermic injections of morphia (gr. $\frac{1}{8}$) and atropine (gr. $\frac{1}{200}$) have proved very satisfactory, generally giving two or three hours or more of refreshing sleep, the patient when awake feeling comfortable and rid of the restless condition previously present. Sulphonal is occasionally given, but has not proved very reliable. Doses of gr. x., xx., or xxx. have in some cases proved effectual, but more often sleep is not procured; and even if the drug has acted as desired the patient is apt on waking not to feel refreshed, and headache is not infrequent.

